# FOS mapping reveals two complementary circuits for spatial navigation in mouse

**DOI:** 10.1038/s41598-024-72272-8

**Published:** 2024-09-11

**Authors:** Edyta Balcerek, Urszula Włodkowska, Rafał Czajkowski

**Affiliations:** Nencki Institute of Experimental Biology, Polish Academy of Sciences, Warszawa, Poland

**Keywords:** Spatial memory, Hippocampus

## Abstract

Here, we show that during continuous navigation in a dynamic external environment, mice are capable of developing a foraging strategy based exclusively on changing distal (allothetic) information and that this process may involve two alternative components of the spatial memory circuit: the hippocampus and retrosplenial cortex. To this end, we designed a novel custom apparatus and implemented a behavioral protocol based on the figure-8-maze paradigm with two goal locations associated with distinct contexts. We assessed whether mice are able to learn to retrieve a sequence of rewards guided exclusively by the changing context. We found out that training mice in the apparatus leads to change in strategy from the internal tendency to alternate into navigation based exclusively on visual information. This effect could be achieved using two different training protocols: prolonged alternation training, or a flexible protocol with unpredictable turn succession. Based on the c-FOS mapping we also provide evidence of opposing levels of engagement of hippocampus and retrosplenial cortex after training of mice in these two different regimens. This supports the hypothesis of the existence of parallel circuits guiding spatial navigation, one based on the well-described hippocampal representation, and another, RSC-dependent.

## Introduction

Choosing the optimal strategy for wayfinding and spatial exploration is one of the fundamental tasks for all animals. Maintaining the correct balance between exploiting the already known territories and discovering the new ones is a continuous process, supported by a dedicated network of brain structures. Historically, the research on forming, updating, and maintaining the spatial map has been mostly centered around the hippocampal formation^1–3^. Hippocampus (HPC) harbors place neurons that fire preferentially at certain positions (place fields) within the known environment^4^. The combined activity of place cells provides the actual neurophysiological underpinning of the cognitive map^5^. One of the crucial features of the place neurons is the ability to remap in a novel environment, a process that is manifested by a complete rearrangement of spatial firing preferences for the entire hippocampal population^6^. This effect can be achieved experimentally by physical transfer to a new place, but also by a drastic and rapid rearrangement of salient external cues (“teleportation”)^7^. A number of distinct, non-overlapping maps can be recorded in a laboratory setting^8^.

Recent studies suggested that structures other than the HPC might also participate in the process of spatial mapping^9^, with various degrees of independence. One of the heavily investigated but still underappreciated regions is the retrosplenial cortex (RSC)^10^. It connects extensively with the hippocampal formation, but also independently with other areas, including the anterior cingulate, visual, and parietal cortices^11^. We have previously contributed to mapping the relevant connectivity of the RSC and revealed a direct functional input from this structure into the deep layers of the medial entorhinal cortex (MEC)^12,13^. These layers serve as the output of the MEC-HPC loop, therefore any information directly modulating their activity might affect the outcome of hippocampal processing. We recently confirmed that the HPC and RSC input converge on the same population of MEC neurons^14^, suggesting the existence of two parallel circuits sharing the same output^15^. Others have shown that the hippocampus might exert an inhibitory effect on the RSC activity via direct inhibitory projections from CA1 and subiculum, preventing competition between the two structures^16^. We have also shown that a measurable behavioral output in a spatial exploration paradigm could be produced by a range of alternative brain networks. In a seemingly homogeneous population of animals, different spatial strategies might spontaneously emerge and develop^17^.

In this paper we assessed whether mice are able to establish an efficient navigational mode based exclusively on changing distal (allothetic) information. We then asked the question if different learning regimens could lead to similar behavioral outputs and if the training history would be reflected in differences in RSC and HPC activity during the test session. To his end, we designed and implemented an automated, semi-autonomous system for spatial memory tests. It is based on the T-maze paradigm^18–21^, with modifications allowing for reliable dependence on visual cues. Standard T-maze consists of a stem (starting arm) and two perpendicular lateral goal arms (left and right) arranged in the shape of a T. The very first report of the phenomenon of rodent spontaneous alternation comes from Edward Tolman's study from the 1920s^22^. The classical procedure is based on the natural tendency of rodents to explore a novel arm over a familiar one, which prompts them to alternate the choice of the goal arm across repeated trials in the “win-shift” arrangement. Goal arms are baited with rewards to reinforce learning^23^. T-maze can be designed with enclosed or open arms and animals can follow discrete or continuous trials^24^. As the animal has to remember which arm had been visited in the previous trial, those cases utilize rule learning and working memory by forcing recourse to recently acquired information. We intended to check whether this internal alternation tendency can be substituted by learned flexibility allowing unpredictable turn successions and whether animals are able to rely exclusively on visual cues while navigating. To reduce the influence of rule learning and the involvement of working memory we utilized a novel spatial behavioral protocol based on the figure-8-maze paradigm with reinforced cue-location associations. This solution employs a fully automated closed-loop system using visual contexts for guiding behavior. It allows high throughput testing of large cohorts of animals with reduced stress effects as human intervention is minute. We trained two groups of animals (“Alternation” and “Modified”) in two different navigational regimens.

In the “Alternation” group the natural tendency to follow the win-shift rule between the forced and choice phase was always supported by congruent changes in spatial context. We expected that this would lead to strengthening the alternation behavior during acquisition and to possible formation of a habit, with little if any spatial memory component. We regarded the alternation group as the reference control. The “Modified” group had to overcome the discrepancy between the natural alternation behavior and the use of visual cues, since turn directions changed randomly, while context-reward association remained fixed. Under these conditions, a range of outcomes was expected. Using these two different regimens, we trained two groups of animals to perform the task at comparable levels. We then mapped the level of c-FOS protein in the hippocampus and the retrosplenial cortex. *Fos* expression is rapidly induced following host neuron activity leading to structural and functional cell changes which are essential for memory formation^25,26^. Kinetics of the FOS response to relevant external stimuli is transient, with a peak of *c-Fos* mRNA approximately 30 min and c-FOS protein between 90 and 120 min^27^. FOS imaging is therefore widely used as an accessible and reliable method allowing active cells to be identified^15^.

## Materials and methods

### Animals

Fourteen young (12 weeks old) male, wild-type C57BL/6 mice were used for this experiment. One animal was excluded during training due to health issues. Experiments were performed with individually housed mice. Animals were kept on a reversed 12-h light/ dark cycle, with the dark phase starting at 7 am. All behavioral procedures were conducted during the dark phase. Mice had ad libitum access to food and water before the experiment. Along with the beginning of experimental procedures, minimal food restriction (3,5 g daily/mouse) was implemented in order to raise animals' motivation for the food reward. Average daily body weight was monitored for each animal and never dropped below 90% of initial body weight.

All experiments were approved by the 1st Local Ethical Committee in Warsaw (code: 1397/2022). The experimental protocol followed the European Communities Council Directive and The Law on the Protection of Animals Used for Scientific or Educational Purposes.

### Apparatus

**Fig. 1:**
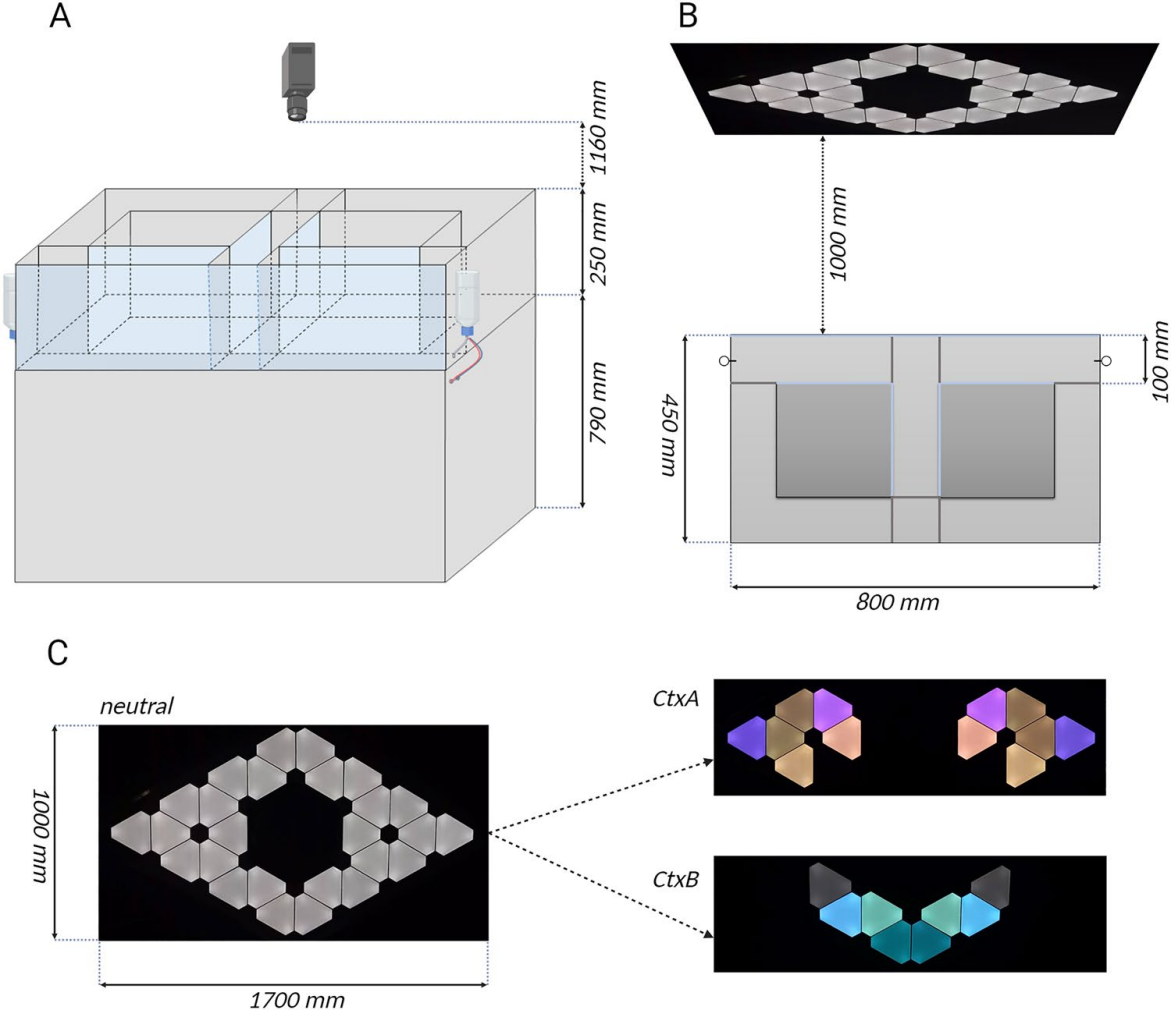
Figure-8-maze apparatus (see text for details) (**A**) Schematic front view with camera and reward dispenser locations. (**B**) Schematic top view with LED panels placed 1000 mm ahead of the transparent, front wall showing neutral context used for habituation protocol as well as for starting and ending of each daily session of behavioral procedure. Transparent walls are light blue colored. All inner doors are pneumatically movable in order to enable continuous movement of the animals and to force a desired turn direction when necessary. (**C**) Realistic view of displayed visual cues. Neutral context (left) showing the full array of triangular LED panels, CtxA (right, top) associated with right turn, CtxB (right, down), associated with left turn.

A fully automated figure-8-maze apparatus, (external dimensions of the enclosure: 80 × 45 cm, wall height 25 cm) was designed and in-house manufactured (Fig. 1A). All front walls and the stem walls were made of transparent plexiglass, and all other walls and the floor were opaque. The corridor width was 10 cm. The length of the stem section was 25 cm, the length of each arm was 35 cm. Seven pneumatically operated doors were installed to enable continuous movement of the animals and to force a desired turn direction when necessary (Fig. 1B). Visual information was presented using an array of triangular LED panels (Aurora system, Nanoleaf). Two different visual contexts (Fig. 1C) were displayed in front of the maze. Both contexts varied in terms of colours and spatial arrangement of LED panels. Each context was associated with a specific turn direction. Sweet condensed milk solution (approx. 10 μl) was used as the food reward. The reward was administered at the front corners from dispensers with electromagnetic valves via 18G blunt needles.

### Habituation

For all behavioral procedures mice were handled for at least 14 days prior to the onset of training. During the last 7 days, handling sessions were designed to mimic the final experimental protocol, including appropriate light and sound changes, conditions of the experimental room, and getting used to the apparatus. In order to completely eliminate the novelty stress associated with the reward, mice were exposed to sweet reward (condensed milk) in the home cage for 4 min/day on four consecutive days. Milk was applied through the same dispensers as used in the apparatus during training. Each time the mouse approached the dispenser, it received a drop of milk (approximately 10 μl). When all animals developed a stable approach behavior towards the reward immediately after inserting the dispenser, the activity was completed. A few hours after milk stimulus exposure mice were habituated to the figure-8-maze apparatus, where subsequent stimuli were introduced gradually. For two consecutive days animals were able to move freely inside the maze for 4 min/day with no door movement and the reward was automatically provided each time the mouse approached the dispenser area. During the next two days mice were placed in the apparatus for 4 min/day with some (5 out of 7) motorized doors activated and the reward given only once per specific arm entry. Each exposure to the maze apparatus prior to the onset of training was accompanied by the neutral context displayed on LED panels, as the only light source (Fig. 1C).

### Experimental protocol

**Fig. 2:**
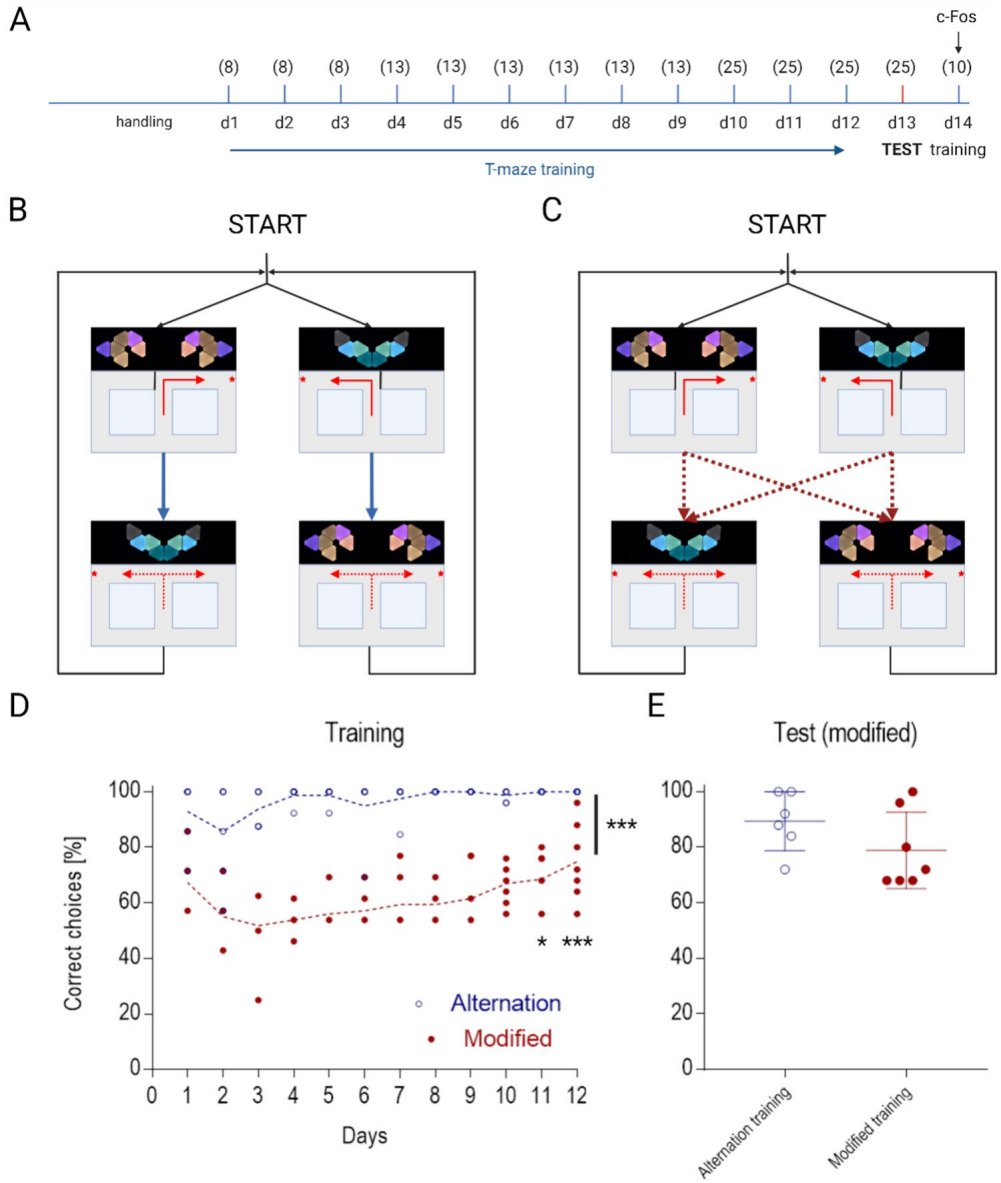
Behavioral experiment. (**A**) Timeline representation of the experimental design (see Methods for details). Numbers in brackets indicate subsequent numbers of forced-choice trials on each day of spaced training. The final number of right and left turns (corresponding with visual cue) was equal on each day. (**B**) Schematic representation of “Alternation” training strategy. A rigid order of turns was imposed. (**C**) Schematic representation of “Modified” training strategy. Any sequence of turn directions was possible between forced and choice phase while maintaining an equal proportion of every possible sequence. (**D**) Learning curve for “Alternation” and “Modified” groups. Data points show the percentage of rewarded decisions (congruent with the displayed context) by individual animals in each session. Significant effects labeled: (***) training effect, F(1,11) = 1.499, *P* < 0.0001, two-way repeated measures (RM) ANOVA; training day for modified group: (*) day 11 (*P* = 0.023), (***) day 12 (*P* = 0.0002), post hoc Tukey test. (**E**) Test session in “Modified” layout. Data points show the percentage of decisions congruent with the displayed context.

After the habituation period mice underwent 12 days of spaced training (Fig. 2A-C). Each trial consisted of two phases with the mouse continuously passing through the figure-8-maze. Each trial began with the forced phase, with the animal starting in the center zone, entering the single available arm of the figure-8-maze, and immediately after collecting the reward returning to the center zone. Mice were prevented from retracting into the choice arm by closing doors and always continued the only available way via the connecting arm back to the base of the T. The second phase of the trial (choice phase) started with both arms open so that the animal could make a direct choice and decide which arm to enter. All inner doors were pneumatically movable in order to enable continuous loop movement of the animals and to force a desired turn direction when necessary. The contact with the experimenter was reduced only to exposure before and after the completion of the daily session. Both runs were rewarded, but milk was given only in the correct arm, according to the visual context displayed in front of the maze. On trials with incorrect choice responses, no reward was provided and the mouse was required to continue along the connecting arm, re-enter the central zone, and make the correct choice on the following forced trial. Contexts were defined specifically for left and right turns respectively (Fig. 1C) and displayed during both runs (forced and choice) allowing navigating based on external directions at each run. Each animal underwent a progressively increasing number of forced-choice trials per day. Training started with 8 trials per day and reached the full number of trials—25 on the 10th day of training. Two groups of animals (“Alternation” and “Modified”) were trained in two different navigational regimens. Alternation training was based on a repetitive scheme of contexts displayed in alternating order. This strategy takes advantage of the natural tendency of rodents to explore unvisited territory^22^. Each forced run was immediately followed by the choice run with the changing of visual context. The correct response for the animal was to choose the opposite arm from the one it visited on the previous (forced) run (win-shift strategy) (Fig. 2B). The order of forced/choice trials was pseudorandom and didn't vary over days of training, simultaneously maintaining a balance of 25 left and right locations within the daily session for both alternation and modified layout. The full (25-trial) training sequence was as follows (forced/choice). Alternating group: L/R R/L R/L L/R L/R R/L L/R R/L R/L L/R R/L L/R L/R R/L L/R R/L R/L L/R R/L L/R R/L L/R R/L L/R R/L. Modified group: L/R L/L R/L R/R L/R L/L R/L R/R L/R L/L R/L R/R L/R R/R L/L L/R R/L L/L R/R R/L L/R L/L R/L R/R L/R. Modified training requires flexible response to visual stimuli. In half of the trials, the schematic, alternating sequence described above was replaced with trials in which the system displays identical context in both forced and subsequent choice runs. The rewarded response is for the animal to choose the same arm it had visited on the previous (forced) run. Such complex trials (with the same context displayed in both forced and choice runs) were programmed among alternating trials with a frequency of 50% of all trials (Fig. 2C). Additionally, besides the within-trial “win-stay” decisions, the automated figure-8-maze continuous movement gave the possibility to force the re-entrance into the same arm between trials (e.g. R/L L/R- left arm in the choice phase is followed by the left arm rewarded in the forced phase of the next trial). Such an approach exposes mice to subsequent identical contexts even if it does not depend on their own decisions. As a result, the total number of reentrances to the same arm was counterbalanced between the two groups. The experimental design is summarized in Fig. 2A-C. We designed a closed-loop system in which the position of the animal was tracked. After the animal made a choice, the doors to both arms were closed and the reward was dispensed only when the chosen arm was the correct one. The final number of right and left turns (corresponding with visual cue) was equal each day. Both training groups were tested on the 13th day of the experiment in an identical manner. The test was very similar to the strategy of “Modified” training with one exception: all 25 forced-choice trials of flexible response to changing visual cues were rewarded to avoid extinction.

### FOS induction

In order to induce the expression of FOS protein, 24 h after the behavioral test, each animal was subjected to 10 trials (20 subsequent runs) in the “Modified” layout (Fig. 2A). Ninety minutes after the beginning of the task, each animal was quickly sedated with a mixture of isoflurane (5%) in conjunction with pure oxygen, followed by intraperitoneal administration (30G needle) of ketamine (75 mg/kg) and medetomidine (0.5–1 mg/kg) mixture. Then mice received an overdose of sodium pentobarbital (150 mg/kg) and were perfused transcardially with phosphate-buffered saline (0,1 M PBS, 50 ml, RT) with heparin (10 000 units/l) followed by paraformaldehyde (PFA) perfusion (4% paraformaldehyde solution in 0.1 M PBS, 150 ml, 4 °C). The brains were collected and stored for 24 h in PFA, followed by a sucrose solution (30% sucrose in 0.1 M PBS). Brain slices were cut coronally (40 µm) in a cryostat and subjected to histological analysis.

### Immunostaining

In order to detect FOS protein, free-floating coronal brain sections were first washed for 3 × 10 min in 0.1 M PBS (40 rpm). Background staining (activity of aldehyde group) was diminished by 15 min incubation in 1% NaBH_4_ in 0.1 M PBS (2 rpm) followed by subsequent washes in 0.1 M PBS (1 × 10 min, 3 × 5 min, 30 rpm). Endogenous peroxidase activity was blocked by incubating the brain sections in a solution of 3% hydrogen peroxide and 70% methanol in distilled water for 10 min (10 rpm), followed by four subsequent washes in 0.1 M PBS, 5 min each (30 rpm). Sections were blocked in 5% normal goat serum (NGS) in 0.3% PBST (phosphate-buffered saline solution with a 0,3% concentration of triton x-100) for 4 h and incubated 48 h at 4 °C with solution of primary c-FOS rabbit polyclonal antibody diluted 1:1000 (Synaptic Systems, Göttingen, Germany, cat. nr: SYSY 226 008) in 0.3% PBST with 5% NGS. Incubation was stopped with 0,3% PBST / 5% NGS (5 min, 30 rpm), and sections were washed three times in a solution of 0,3% PBST / 2% NGS, 10 min each (30 rpm). Then a secondary biotinylated goat anti-rabbit antibody was applied in PBST / 2% NGS (1:200; Vectastain Elite Kit, Vector Laboratories, Burlingame, CA, USA) (75 min, RT, 10 rpm). The sections were then washed (5 × 5 min, 20 rpm) and processed with avidin-biotinylated horseradish peroxidase complex (Vectastain Elite Kit, Vector Laboratories) in 0.3% PBST for 1 h at room temperature (2 rpm), followed by subsequent washes in 0.3% PBST (4 × 5 min, 15 rpm). The reaction was visualized using diaminobenzidine (DAB, Vector Laboratories), enhanced by nickel salt, and was stopped by PBS washes (4x). Sections were mounted on glass slides and left overnight. Sections were then further dehydrated in graded alcohol concentrations (ethanol), defatted by xylene, and coverslipped with a water-free mounting medium (Entellan).

### FOS cells identification

Digital images were captured with an Olympus VS110 microscope. High-resolution scans of the whole brain slice specimen were taken. To acquire the best precision in selecting the region of interest digital histological sections were overlaid with relevant transparent atlas figures shaping the frame of structures borders. Frames of brain coronal borders were created in the GNU Image Manipulation Program (Free Software Foundation). Image analysis was performed with the ImageJ platform followed by custom-made semi-automated scripts detecting locations of c-FOS positive cells. *c-Fos* expression was reflected by the density of c-FOS immunopositive nuclei that were labeled above a set threshold. The threshold was determined individually for each region of interest: rRSA, cRSA, rRSG, cRSG, dCA1, dCA3, dDG, and was kept the same among all mouse samples. Brain sections were identified according to the atlas of Paxinos and Franklin^28^. For each brain, counts were taken from seven anterior–posterior levels centered around the target AP coordinate (rostral part of RSC (rRSA, rRSG) − 1.50 mm; caudal part of RSC (cRSA, cRSG) − 2.90 mm; dorsal HPC (dCA1, dCA3, dDG) − 1.50 mm from bregma respectively). The total number of c-FOS- immunopositive nuclei from both right and left sides was considered. To calculate the density [cells/mm^2^] of c-FOS positive cells in each area, the number of c-FOS stained cells in a given field was counted and divided by the area that was occupied by the given field.

### Data analysis

All results were analyzed by researchers unaware of the exact experimental conditions. Datasets were aggregated in Excel and further analyzed in Prism v7.01 (GraphPad Software). To compare learning progress in the Alternation and Modified groups, two-way repeated measures ANOVA was applied, with the post-hoc Tukey test to analyze individual time points. To compare the test performance, t test was applied. For c-FOS density comparison Holm-Sidak multiple t test was used. All values are provided as means with standard deviation indicated.

## Results

We first verified the ability of mice to perform the visually guided version of the figure-8-maze task in the automated apparatus following two different training protocols. In both versions of the task, mice were expected to associate the displayed spatial context with the rewarded turn direction (see Methods for details). In the “Alternation” training scheme, apart from the spatial guidance, the win-shift strategy was imposed on the animals, with the reward location changing between the forced and choice phase for each trial. Accordingly, context was switched between the forced and choice phase, always remaining congruent with the turn direction. In this case, contextual information was additionally supported by the rule learning. As expected, mice (n = 6) acquired the task within no more than three sessions and remained at the asymptotic level for the rest of the training (accuracy in the last training session: 100%, Fig. 2D). In the “Modified” version, with no predictable alternation, the win-shift strategy was correct only in 50% of the trials. In the remaining half, the win-stay strategy was imposed and the animals had to reenter the previously visited arm in order to collect the reward. For each phase of each trial, the goal location was aligned with a specific visual context, therefore the ability to flexibly suppress the win-shift strategy and to rely solely on the contextual information was necessary for successful learning. Under such a regimen, mice (n = 7) initially performed around chance level. The apparent lack of consistent reinforcement led to an initial decrease in performance, reaching a minimum at Session 3 (average accuracy: 51.8%, s.d. = 12.37). However, over the course of Sessions 4–12 the group gradually progressed and at the conclusion of the training phase, some animals in this cohort improved to almost match the performance of the “Alternation” group (average accuracy in the last training session: 74.9%, s.d. = 12.95, Fig. 2D). As expected, during acquisition, win-stay trials were more difficult to overcome compared with win-shift trials, even if supported with subsequent learning success in some individuals (see Fig. S1, supplementary data). Overall, the two-way repeated-measures (RM) ANOVA, group (alternation, modified) × time (days 1–12) showed significant effects for group: F(1,11) = 1.499, *P* < 0.0001, time: F(11,121) = 3.839, *P* < 0.0001 and interaction: F(11, 121) = 1.878, *P* = 0.0486. Post hoc Tukey multiple comparisons test of the modified group showed a significant effect of training on day 11 (*P* = 0.023, 95% CI = − 32.36, − 1.213) and day 12 (*P* = 0.0002, 95% CI = − 38.64, − 7.499). We then tested the ability of both training groups to solve the task in the “Modified” layout, with 50% of the trials requiring reentry to the previously visited arm. During the test, both arms were rewarded in the choice trial to avoid memory extinction. As expected, in the “Modified” group we observed performance similar to the last training session (average accuracy: 78.8%, s.d. = 13.8, Fig. 2E). Unexpectedly, the “Alternation” group, despite following only the schematic, win-shift protocol during training, also performed at a high level in the “Modified” test (average accuracy: 89.3%, s.d. = 10.6, Fig. 2E) and no statistical difference between groups was observed with two-tailed t test (t = 1.511, d.f. = 11, *P* = 0.159).

**Fig. 3:**
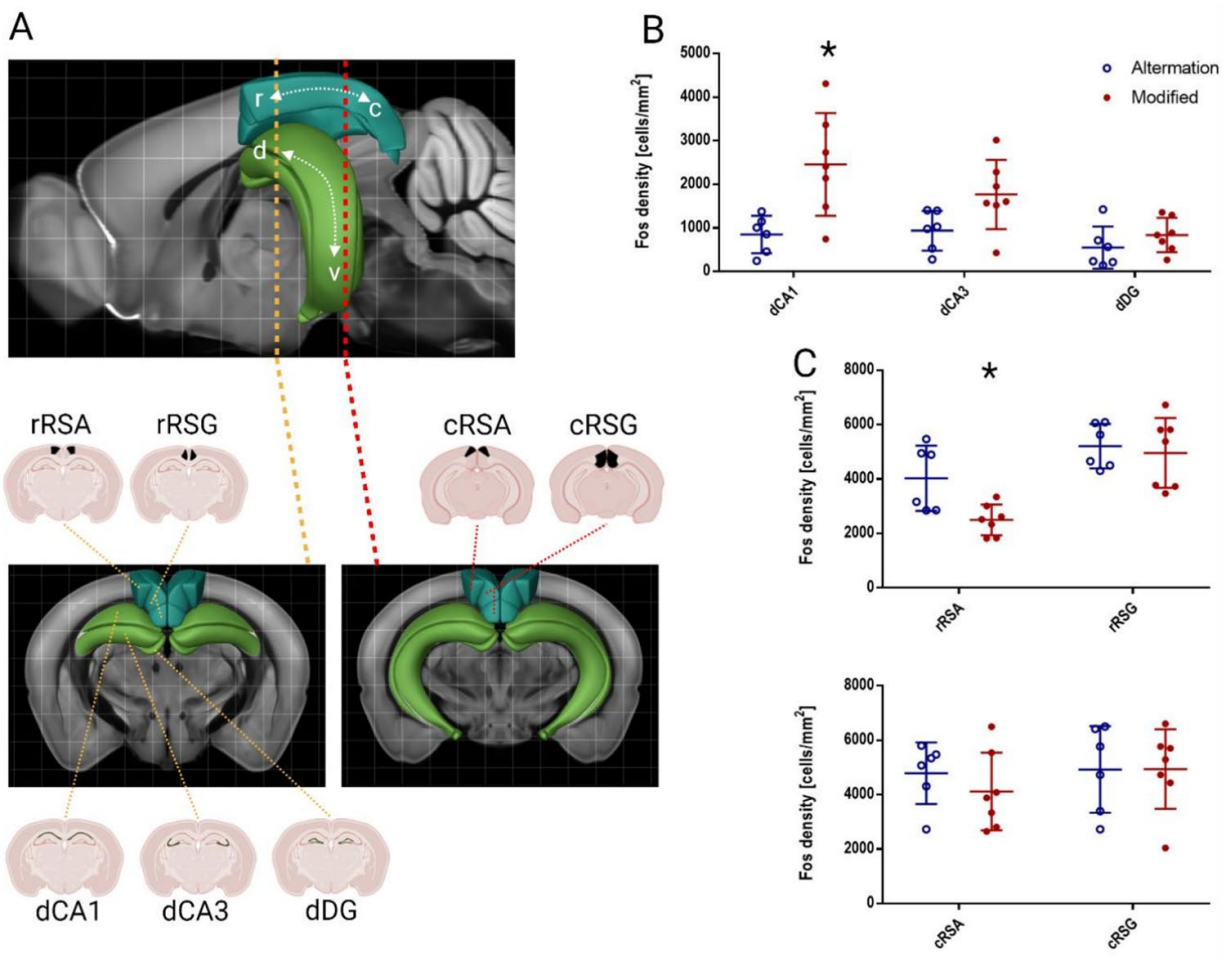
Comparison of c-FOS-positive cell density between training protocols across brain regions. (**A**) Visualization of the position of central slices for each region of interest in the mouse brain using Allen Brain Atlas. In the upper panel, the hippocampus and retrosplenial cortex are marked with colors, and the rostro-caudal position of the slices is indicated. The lower panel shows the position of ROIs for c-FOS positive cell density collection on the coronal slices. (**B**) Density of c-FOS immunopositive cells in the ROIs of the dorsal hippocampus, compared between both experimental groups. A statistically significant difference between training protocols has been revealed for CA1, where c-FOS protein level proved to be higher in mice trained in the “Modified” protocol (*P* = 0.027, Holm-Sidak multiple t test). A similar trend (however, with no statistical significance, *P* = 0.088) was observed for CA3. (**C**) Group differences of activation of the rostral part of granular (rRSG) and agranular (rRSA) subdivisions of RSC. A significant suppression of the c-FOS protein level in the “Modified” protocol was observed for rRSA (*P* = 0.023, Holm-Sidak multiple t test). A similar analysis for the caudal part or RSC shows no visible difference. This result proves the existence of an activation gradient along the rostro-caudal axis for the agranular retrosplenial cortex.

After 24 h we performed another training session (20 subsequent runs) in the “Modified” layout in order to induce *c-Fos* expression in the relevant brain areas responsible for spatial learning. 90 min after the beginning of the task, mice were perfused and brains were processed for FOS immunohistochemistry. We focused the analysis on two relevant regions, previously identified as involved in spatial memory, navigation, and wayfinding (Fig. 3A). We analyzed the dorsal HPC (dentate gyrus, CA1, and CA3 fields) and the RSC (granular and agranular) at two coronal planes (rostral and caudal, see Methods for details). We compared expression levels between mice trained in two learning protocols, but tested in the same conditions with similar behavioral outcomes. Our analysis revealed that the dorsal hippocampus becomes highly activated in the group that was trained in the “Modified” protocol (Fig. 3B). Density of c-FOS immunopositive cells was much higher in the dorsal part of the CA1 field (2456 cells/mm^2^, s.d. = 1178) compared to mice from the “Alternation” group (848 cells/mm^2^, s.d. = 428, adjusted *P* = 0.027, Holm-Sidak multiple t test). A similar trend was observed in the CA3 region, but when adjusted for multiple comparisons, the difference did not reach statistical significance (1765 cells/mm^2^, s.d. = 794 vs. 934.7 cells/mm^2^, s.d. = 457, adjusted *P* = 0.088, Holm-Sidak multiple t test). The dentate gyrus, a subregion of the hippocampal formation usually showing sparse *Fos* expression, does not seem to be more active in the alternation group (549 cells/mm^2^, s.d. = 484) compared to the modified protocol (836 cells/mm^2^, s.d. = 392, adjusted *P* = 0.262, Holm-Sidak multiple t test).

A similar analysis performed for the retrosplenial cortex included two coronal planes (see Methods) since functional gradients have been previously reported for this structure along the sagittal axis. In the “Alternation” group we observed an almost uniformly high activation across RSC subregions (Fig. 3C), with no differences along the rostro-caudal axis (rRSA, 4031 cells/mm^2^, s.d. = 1200, rRSG, 5209 cells/mm^2^, s.d. = 814, cRSA, 4789 cells/mm^2^, s.d. = 1129, cRSG, 4925 cells/mm^2^, s.d. = 1590). In the “Modified” group, the activity of rostral RSA was markedly suppressed (2494 cells/mm^2^, s.d. = 567, adjusted *P* = 0.023, Holm-Sidak multiple t test), while RSG and caudal part of RSA were activated at a similar level as the “Alternation” group (rRSG, 4958 cells/mm^2^, s.d. = 1284, cRSA, 4114 cells/mm^2^, s.d. = 1426, cRSG, 4938 cells/mm^2^, s.d. = 1464).

We also verified the correlation of behavioral response with FOS levels in all brain regions of interest (Fig. S2, supplementary data). Spatial learning ability for individual mice defined as task acquisition (measured by the percentage of correct turns according to the visual cues in the test) did not correspond with the c-FOS level in the “Modified” group. On the other hand, the performance of the “Alternation” group during the test trial was strongly correlated with the FOS level in rostral RSG (Spearman correlation, r = 0.899, adjusted *P* = 0.028).

## Discussion

Studies of spatial learning and navigation based explicitly on the allothetic information have always been considered a technical and conceptual challenge when applied in the mouse model. In this work, we successfully implemented a novel behavioral task that tests the ability of mice to follow the correct path and to retrieve a reward, based solely on the spatial context presented. Although similar automated maze devices were constructed previously^20,21,24,29–31^, and the T-maze protocol has been tested in a plethora of previous studies^19,32–36^, it was always used in the win-shift arrangement, where spatial information merely supported the rule learning. The animals had to remember the given spatial orientation and utilize it to choose the opposite arm during the choice phase. Notably, in such cases, working memory also played an important role in solving the task. We show it is possible to dissociate the spatial memory from the rule learning and to completely eliminate the working memory component, either during the training phase or selectively in the behavioral test. To the best of our knowledge, only one similar attempt has been successfully shown^37^. However, the VR-based model used in this study in combination with an immobilized mouse eliminated active movement and limited the range of practical applications. In our approach, high throughput experiments are possible, with the capability of training and testing up to tens of animals in one day session. This opens a possibility to use it as a broad screen for the mechanisms of spatial memory impairments in the mouse model, similar to the solutions reported before^21^.

Considering the fact that our behavioral test session revealed two populations with matching behavioral performance, we could compare the relative involvement of two key brain regions responsible for spatial memory and navigation: dorsal hippocampus and retrosplenial cortex in task completion. We used the expression patterns of *c-Fos* immediate early gene as a proxy for behaviorally relevant neuronal activation^15^. Two distinct behavioral approaches used for training allowed for within-experiment comparisons, without the need for additional naive control. Despite the difference in training regimens both groups associated the visual cues with the turning direction and utilized acquired visual strategies during the test procedure. Interestingly, there was a marked difference in the hippocampal activity in the group that followed the flexible learning and working memory-independent regimen already during training (“Modified” group) with no concomitant distinction in test success rate between groups. It was particularly prominent in the CA1 region, although a similar trend could be observed along the entire trisynaptic pathway.

The limited scale of performed analysis does not allow for definite answers, but it could be speculated that the hippocampal formation is activated when spatial navigation in the known environment is driven by the need for flexible location decisions, determined by competing or overlapping extra maze cues. A similar phenomenon has been demonstrated before, and NMDA-dependent plasticity in the dentate gyrus and dorsal CA1 was shown to be the molecular mechanism beyond the observed effect^38,39^. In the case of the “Modified” group, mice were forced to use flexible navigation from the onset of learning, since in order to get the reward they were following randomly changing contexts. Their attention was highly focused on extra maze cues, as there were sequence arrangements requiring consecutive voluntary entry into the same arm. It is possible that this high attention necessity may drive different levels of plasticity associated with learning and can be the reason for stronger hippocampal engagement. On the other hand, this difference may also stem from the fact that the “Modified” group received fewer reinforcements during training. Despite identical exposure to the environment, the experience of the two groups was not perfectly matched. It is worth mentioning that during test protocol and sessions preceding terminations rewards were administered on both sides, to avoid extinction and to prevent affecting FOS level by the imbalance of appetitive stimulations.

Apart from differences in hippocampal activation, in the “Modified” group, the activity of the rostral part of agranular retrosplenial cortex (RSA) is diminished, as compared to the “Alternation” group. This observation is in accordance with the previously reported negative feedback between the output fields of the hippocampal formation and the RSC. These direct inhibitory connections were first described on the anatomical level^16^. Functional studies confirmed these findings and revealed additional disynaptic inhibitory input^40–42^. According to these reports, the projections from CA1 and subiculum terminate in the granular retrosplenial area (RSG), but the region affected in our case is RSA. This effect might be due to the interactions between the two regions, as described in^43^. We also observed a gradient of activity along the rostro-caudal axis of the RSA. This confirms previously observed differential roles for rRSC and cRSC^37,44^, with the caudal part encoding the features of the environment, and the rostral area responsible for motor planning. The existence of a negative balance between HPC and RSC supports the concept that these structures may act competitively to drive spatial behavior. Under the conditions where the hippocampus is engaged, it suppresses RSC in order to avoid conflicting behaviors. RSC is a likely candidate for harboring the alternative memory center. It receives sensory inputs from the visual cortex^9^, and engages in an independent thalamocortical circuit^45^. The presence of the molecular signature of a memory engram in this structure has been previously described by us and others^46,47^. Notably, the RSC activity in the “Alternation” group is constantly high in all analyzed subregions, while the hippocampus is relatively inactive under these conditions. Animals in this group have access to exactly the same variety of external cues but during training, it is displayed in sequence preserving the natural tendency to alternate. We expected that the acutely established win-shift protocol would promote alternation choices also on the test day. Instead, we observed that while employment of naive alternation capabilities benefitted performance during acquisition, it did not impair learning of visual cue-location associations, as mice performed at the same, high level as the “Modified” group. Interestingly, the activity of rostral RSG strongly correlated with behavioral performance of individual animals in this group, suggesting a pivotal role of this structure in memory acquisition under non-flexible, repetitive regimen. A number of recent reports provide ample evidence that RSC could support spatial memory and navigation by itself. It encodes the animal's position^48,49^, trajectory progression^50^, head-turning direction^51,52^, and goal location^53^ but also a number of unique spatial correlates^54^, including motion- and direction-independent heading signal^55^ which suggests the existence of purely egocentric spatial code^56^. RSC could therefore shift rapidly between allocentric and egocentric spatial representations in order to support navigation. Indeed, lesion and inactivation data suggest the involvement of RSC in a number of spatial tasks, but its disruption rarely leads to a complete deficit^33,46,57–59^. Based on our current results, we postulate that a specific training protocol optimized for RSC engagement could minimize the involvement of the hippocampus and allow for easy dissecting of the alternate spatial memory circuits.

## Supplementary Information

Supplementary Information.

## Data Availability

The datasets used and analysed during the current study available from the corresponding author on reasonable request.
