# Supplementary material for: FOS mapping reveals two complementary circuits for spatial navigation in mouse
Source: Sci Rep. 2024 Sep 11;14:21252. doi: 10.1038/s41598-024-72272-8 (PMC11391074; doi:10.1038/s41598-024-72272-8)
Supplement: Supplementary file 1 — Supplementary Information. [file 41598_2024_72272_MOESM1_ESM.pdf]

Supplementary information

FOS mapping reveals two complementary circuits for spatial navigation in mouse.

Edyta Balcerek, Urszula Włodkowska, Rafał Czajkowski

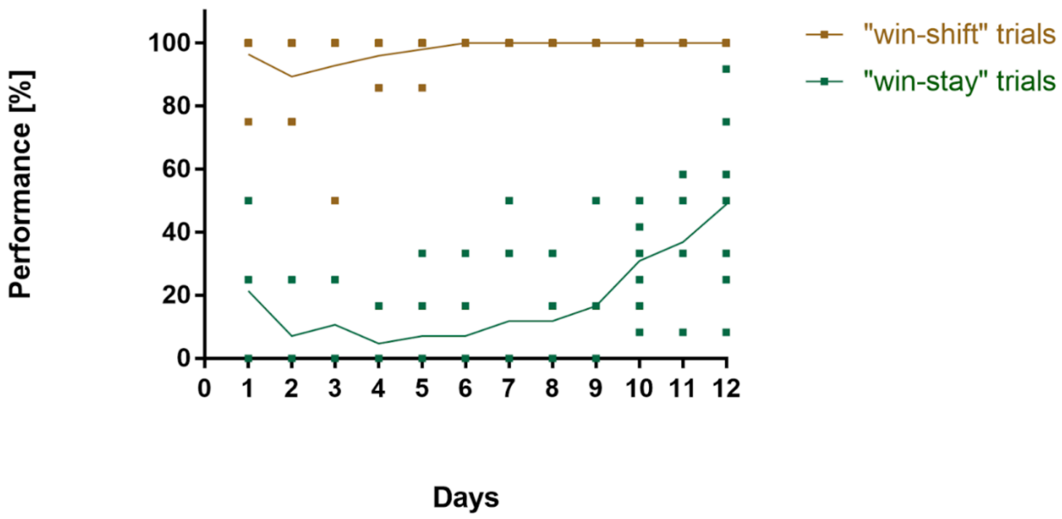

Supplementary Fig. 1. Learning curves for “Modified” group showing the “win-stay” and “win-shift” trials. Data points show the percentage of rewarded decisions (congruent with the displayed context) by individual animals in each session.

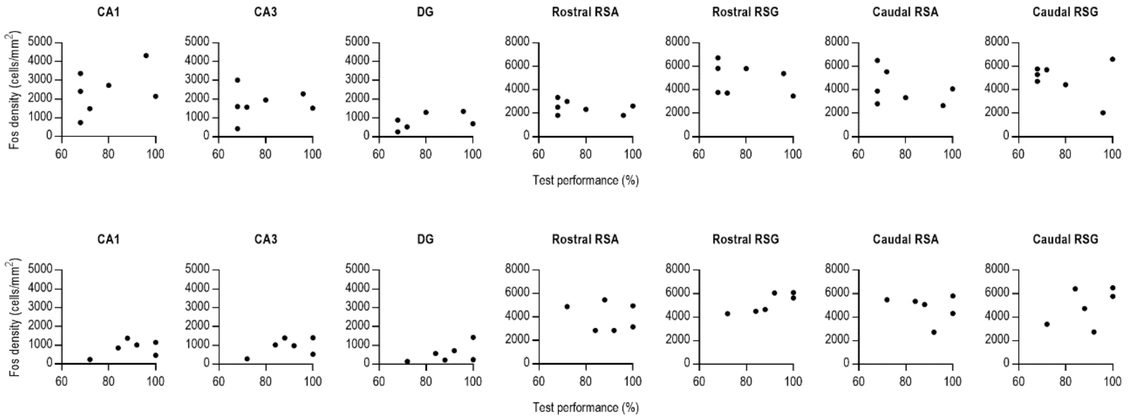

Supplementary Fig. 2. Correlations between behavioral performance during test trial and FOS expression in analyzed brain regions. Significant correlation has been found for rostral RSG in Alternation group (Spearman correlation, r=0.899, adjusted p=0.028).
